# Treatment of Chronic Lymphocytic Leukemia in the Personalized Medicine Era

**DOI:** 10.3390/pharmaceutics16010055

**Published:** 2023-12-29

**Authors:** María Del Mar Sánchez Suárez, Alicia Martín Roldán, Carolina Alarcón-Payer, Miguel Ángel Rodríguez-Gil, Jaime Eduardo Poquet-Jornet, José Manuel Puerta Puerta, Alberto Jiménez Morales

**Affiliations:** 1 Servicio de Farmacia, Hospital Universitario Virgen de las Nieves, 18014 Granada, Granada, Spain; mariadmar157@gmail.com (M.D.M.S.S.); aliciamartinroldan@gmail.com (A.M.R.); alberto.jimenez.morales.sspa@juntadeandalucia.es (A.J.M.); 2 Unidad de Gestión Clínica Hematología y Hemoterapia, Hospital Universitario Virgen de las Nieves, 18014 Granada, Granada, Spain; miguel.rodriguez.gil.sspa@juntadeandalucia.es (M.Á.R.-G.); josem.puerta.sspa@juntadeandalucia.es (J.M.P.P.); 3 Servicio de Farmacia, Hospital de Dénia Marina Salud, 03700 Dénia, Alicante, Spain; jaime.poquet@marinasalud.es

**Keywords:** chronic lymphocytic leukemia, genetic alterations, inhibitors of Bruton tyrosine kinase, TP53 mutation, immunoglobulin heavy chain variable region mutated

## Abstract

Chronic lymphocytic leukemia is a lymphoproliferative disorder marked by the expansion of monoclonal, mature CD5+CD23+ B cells in peripheral blood, secondary lymphoid tissues, and bone marrow. The disease exhibits significant heterogeneity, with numerous somatic genetic alterations identified in the neoplastic clone, notably mutated TP53 and immunoglobulin heavy chain mutational statuses. Recent studies emphasize the pivotal roles of genetics and patient fragility in treatment decisions. This complexity underscores the need for a personalized approach, tailoring interventions to individual genetic profiles for heightened efficacy. The era of personalized treatment in CLL signifies a transformative shift, holding the potential for improved outcomes in the conquest of this intricate hematologic disorder. This review plays a role in elucidating the evolving CLL treatment landscape, encompassing all reported genetic factors. Through a comprehensive historical analysis, it provides insights into the evolution of CLL management. Beyond its retrospective nature, this review could be a valuable resource for clinicians, researchers, and stakeholders, offering a window into the latest advancements. In essence, it serves as a dynamic exploration of our current position and the promising prospects on the horizon.

## 1. Introduction

Chronic lymphocytic leukemia (CLL) or small lymphocytic lymphoma (SLL), the most common leukemia in adults, is characterized by the expansion of monoclonal CD5+CD23+ B cells in peripheral blood (PB), lymphoid tissues, and bone marrow. CLL and SLL share pathology but have different manifestations based on the location of abnormal cells, with CLL in the blood and SLL in lymph nodes. The age-adjusted incidence is 4.9 per 100,000 inhabitants yearly, peaking at a median age of 70, affecting more males than females [1,2,3,4].

Recent trends reveal an increased detection of early stages, allowing close monitoring for asymptomatic cases. CLL is often diagnosed during routine medical visits when a complete blood count shows an elevated lymphocyte count. If the expansion of lymphocytes continues, a flow cytometric analysis is conducted to check the number of CD5+CD19+ B cells, looking for an increased count of cells expressing specific markers [5].

During this asymptomatic phase, clinical history might not reveal much, but some patients may report weight loss, lethargy, night sweats, and complaints of “swollen glands”. Physical examination mainly focuses on identifying swollen lymph nodes, enlarged spleen or liver, and signs of low red blood cells or platelets [5].

**Figure 1:**
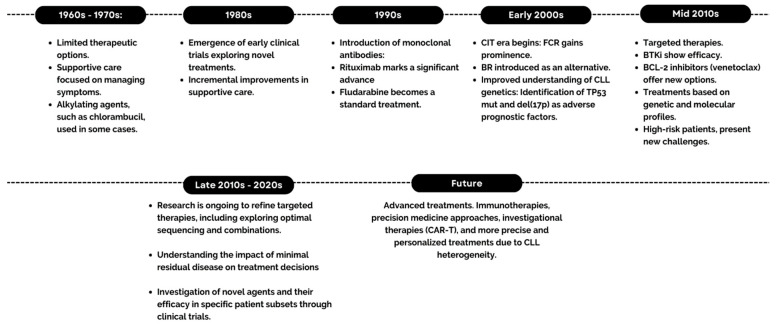
Timeline of the evolution of CLL treatments.

As shown in Figure 1, the evolution of CLL treatment has witnessed significant advancements over the years. Traditionally, CLL management relied on conventional chemotherapy, often utilizing alkylating agents and purine analogs. In 2014, initial treatment was determined by age and comorbidities. Younger patients (<65 years) received chemoimmunotherapy (CIT) like fludarabine, cyclophosphamide, and rituximab (FCR), while those aged 65–75 were given bendamustine and rituximab (BR). Patients over 75 or with significant comorbidities received single-agent chlorambucil, with or without anti-CD20 monoclonal antibody treatment. Therapies targeting B-cell receptor (BCR) signaling have revolutionized CLL treatment, expanding options for high-risk patients with limited previous choices [6,7].

Patients with CLL fall into categories ranging from those minimally affected and not requiring therapy to those with aggressive diseases necessitating immediate treatment. Therapy is now reserved for those with active or symptomatic disease or advanced Binet or Rai stages. Options include venetoclax with obinutuzumab (VO), monotherapy with Bruton tyrosine kinase inhibitors (BTKi) (ibrutinib, acalabrutinib, and zanubrutinib), or CIT. On the other hand, patients with 17p deletion (del17p) or TP53 mutation (TP53mut) resistance to chemotherapy are treated with targeted agents [8,9]. This review aims to approach the therapeutic management of CLL patients from the point of view of personalized medicine. For this purpose, key recommendations from the main expert guidelines for the management of this patient group will be explored, with the treatment of refractory patients strongly influenced by prior treatment and TP53, del17, and immunoglobulin heavy chain variable region mutations (IGHVm) [8,9]. Finally, investigations into new therapeutic strategies for future improvements in CLL treatment outcomes will be highlighted.

## 2. Materials and Methods

For the following literature review, a comprehensive search was conducted in the PubMed and Web of Science databases for articles published within the last decade. Keywords related to CLL, treatment, genetics, mutations, prognosis, and therapy were used in various combinations. In addition, the citations of selected articles were included as supplementary sources. Articles published in English and Spanish were considered, with no restrictions on article type (clinical trials, original articles, reviews, etc.) or population size.

## 3. Pathogenesis

Advancements in our comprehension of the genetics and biology of CLL reveal its considerable heterogeneity. This enhanced understanding allows for a more profound insight into the diverse cell types that could serve as the origins of this malignancy, as well as the genetic elements associated with its pathogenesis. Over time, various cell types have been proposed as potential sources of CLL, building on an evolving comprehension of B-cell biology and differentiation.

CLL may initiate in the stem cell phase, resulting in an increased proportion of polyclonal pro-B cells. This progression may lead, over time, to the development of monoclonal or oligoclonal CD5+ B-cell populations bearing resemblance to monoclonal B-cell lymphocytosis (MBL). Through the acquisition of genetic and epigenetic alterations, hematopoietic stem cells might exhibit a fate bias towards the B-cell lineage. Subsequent antigenic stimulation could then drive the selection and expansion of mature B cells, culminating in the formation of oligoclonal populations. The causes of genetic and epigenetic variations are still unknown [10].

The identification of clonal rearrangements in immunoglobulin genes, coupled with the expression of distinct cell surface markers, has confirmed that CLL originates from a mature B cell. This B cell is notably characterized by low expression levels of B cell markers, including surface membrane immunoglobulins, CD19, and CD20. Additionally, it exhibits positivity for the expression of CD23 (also recognized as FcεRII, a marker present in B cells and dendritic cells) and the antigens CD200 and CD5 [11].

IGHVm CLLs originate from CD5+CD27+ B cells of the post-germinal center, which are transcriptionally like memory B cells and are most likely derived from CD5+CD27− B cells that have undergone the post-germinal center reaction. On the other hand, immunoglobulin heavy chain variable region unmutated (IGHVum) CLLs appear to arise from pre-CG CD5+CD27− B cells, which may be derived from naïve B cells or a separate lineage of precursor B cells. B-cell receptor stimulation, additional genetic and epigenetic abnormalities, and microenvironmental factors will contribute to the precursors of CLL, MBL, and frank monoclonal CLL [10,12].

### 3.1. Genetic Alterations

Several genetic features underlying the clinic-biological heterogeneity of CLL have been described, including immunogenetic features and somatic genetic alterations of the neoplastic clone. The genomic changes in CLL have been extensively investigated using traditional molecular cytogenetics as well as comprehensive approaches such as whole-exome sequencing and whole-genome next-generation sequencing (NGS) [13,14].

Approximately 80% of patients have genetic mutations for del(13q), del(11q), del(17p), or trisomy 12. In contrast, a smaller percentage of patients, around 10–20%, have a more heterogeneous low-frequency mutation profile [15]. Massive sequencing techniques have identified several mutations in different genes, with an average of 20 specific mutations detected per case of CLL, which is considered relatively low in relation to other tumors.

TP53mut is identified in 5–10% of CLL cases at the time of diagnosis, but this frequency escalates to 40–50% among refractory patients [16]. Being among the prevalent genetic alterations in various human cancers, TP53 serves both as a prognostic indicator and a target for treatment. TP53 is a tumor suppressor gene responsible for encoding the p53 protein, which plays a proapoptotic role in response to DNA damage. Positioned on the short arm of chromosome 17 (17p) [17], disruption of TP53 leads to heightened resistance to apoptosis induced by DNA-damaging agents, encompassing chemotherapy, thereby affecting the response to such treatments [18]. The most common genetic lesions of TP53 are somatic mutations and del(17p) [19]. More than 5211 different mutations have been observed in 40416 unique samples across 46 different tissue types, indicative of the genetic variability of TP53 [20].

A rare occurrence is the coexistence of del(17p) with MYC aberrations (translocations or gains) in a very limited number of patients, and this combination may be linked to an exceptionally unfavorable prognosis. However, it is crucial to note that existing studies are primarily retrospective cohorts, with most patients already undergoing treatment. Therefore, it is imperative to conduct further assessments of the TP53 mutational status to ascertain whether the poor prognosis is indeed a consequence of the combination of a TP53mut and not only del(17p) with a MYC aberration [21].

Due to the significant clinical impact, guidelines for the International Workshop on Chronic Lymphocytic Leukemia (iwCLL) recommend testing for del(17p) via fluorescence in situ hybridization (FISH) and TP53 mutation status via DNA sequencing prior to initiating treatment. The European Research Initiative on Chronic CLL (ERIC) advocates considering the application of NGS for the analysis of TP53mut. This approach is distinguished by its higher sensitivity compared to the conventional Sanger sequencing method [22].

As mentioned above, CLL can be divided into two molecular subgroups: (i) non-mutated IGHV CLL (approximately 40% of all CLL), reflecting mature B cells that have not undergone the GC reaction and have undergone T-cell independent maturation, and (ii) IGHVm CLL (60% of all CLL), where mature B cells have undergone the germinal centers (GC) reaction and have undergone the somatic hypermutation process [23]. These IGHVm gene CLLs are genetically developed by activation-induced cytidine deaminase (AID). AID also plays a crucial role in IGH rearrangements, specifically in processes such as class switch recombination (CSR) and recombination between switch Mu (Sμ) and the 3′ regulatory region (3′RR) (Sμ-3′RRrec). Given the predominant presence of unswitched CLL B-cells, an investigation into the blockade of IGH rearrangement in CLL was prompted [24]. Patients with somatic IGHVm with <98% germline homology are considered to have a better prognosis. In addition, it is important to emphasize that, as the mutational status of IGHV is stable throughout the course of the disease, it is not necessary to perform this study again [25].

The presence of TP53mut and the mutational status of IGHV guide treatment decisions by influencing the choice of therapeutic agents and the intensity of treatment. Targeted therapies and alternative approaches are often considered for patients with TP53mut due to chemotherapy resistance, while patients with mutated immunoglobulins may have a more favorable response to less aggressive treatment strategies. The individualized approach to treatment based on these molecular characteristics helps optimize outcomes for patients with CLL. Numerous mutations have been documented, and Table 1 delineates the features of the most significant ones. Currently, none of these mutations are factored into treatment decision making.

### 3.2. Microenvironment

The advancement of tumors involves an intricate process governed by the dynamic interaction between tumor cells and the host immune system. The interaction between the modified functionalities of innate and adaptive immune factors plays a pivotal role in the initiation, progression, and response to treatment in CLL [38]. T cells have some anti-tumor activity, particularly Th1 cells that produce interferon gamma (IFN-γ). Furthermore, regulatory T cells (Treg) play a prominent role in tumor pathogenesis. In CLL patients, the number of Treg cells is increased, and they show signs of exhaustion in proliferation and cellular activity [39].

T cells are primarily responsible for cytokine production. Therefore, alterations in the balance of these molecules increase resistance to cell apoptosis or programmed cell death. The main cytokines affected in CLL patients are interleukin 2 (IL-2), interleukin 4 (IL-4), interleukin 6 (IL-6), interleukin 8 (IL-8), interleukin 9 (IL-9), and interleukin 10 (IL-10) [40]. The CLL implications of each of these are summarized in Table 2.

In CLL patients, a macrophage population acquires a pro-tumor phenotype driven by the CLL cells themselves through the secretion of soluble factors (e.g., IL-10, adenosine, and nicotinamide phosphoribosyl transferase) [50].

In these patients, dendritic cells exhibit dysfunctionality, characterized by changes in the cytokine profile, the absence of the maturation antigen CD83, and the co-stimulatory molecule CD80. Additionally, there is an incapacity to initiate appropriate type 1 T cell responses [49].

The tumor microenvironment of the lymphoid niche is highly hypoxic, and the cells are practically adapted to oxygen deprivation [51]. This stimulates the generation of energy through glycolysis by means of HIF1-α-mediated transcriptional control, tightly managing the expression of glycolytic enzymes as well as glucose and lactate transporters. This encourages a regulatory T-cell phenotype by increasing FOXP3 expression, along with PD-1, IL-10, and VEGFA, while reducing IFN-γ [52].

This metabolic adaptation is accompanied by increased production and release into the extracellular space of intermediates and cofactors such as nicotinamide adenine dinucleotide and adenosine triphosphate. High adenosine signaling is widely associated with immunosuppression in cancer through significantly decreased production of IL-10 and IL-6, negative modulation of T-cell and macrophage depletion markers, and reduced expansion of Treg [51,52].

## 4. Diagnosis, Risk Assessment, and Prognosis

The diagnostic criteria for CLL established by the World Health Organization (WHO), iwCLL, the National Comprehensive Cancer Network (NCCN), and the European Society for Medical Oncology (ESMO) are based on the morphology and immunophenotype of neoplastic B-cells. This includes the co-expression of CD19, CD5, and CD23, with weak CD20 and monoclonal surface immunoglobulin expression [53].

However, the current diagnostic criteria have certain limitations, particularly concerning the flexibility in the requirement for the presence or absence of each marker and the required expression level of each marker. According to the WHO definition, CLL/SLL cells typically co-express CD5 and CD23, and flow cytometry reveals that tumor cells express dim surface IgM/IgD, CD20, CD22, CD5, CD19, CD79a, CD23, CD43, and CD11c (weak). CD10 is negative, and FMC7 and CD79b are usually negative or weakly expressed in typical CLL. It is also acknowledged that some cases may exhibit an atypical immunophenotype [54].

The diagnosis of CLL necessitates the presence of ≥5 × 10^9^/L B lymphocytes in the peripheral blood, persisting for a minimum of 3 months. Confirmation of the clonality of these B lymphocytes is crucial and can be achieved by demonstrating immunoglobulin light chain restriction through flow cytometry. Morphologically, leukemic cells identified in the blood smear exhibit characteristics such as small, mature lymphocytes with a narrow cytoplasmic border, a dense nucleus lacking evident nucleoli, and partially aggregated chromatin. Gumprecht nuclear shadows, also known as smudge cells, are commonly observed as cellular debris in association with CLL. A small proportion of larger or atypical cells, including prolymphocytes, may be present alongside morphologically typical CLL cells. A diagnosis of prolymphocytic leukemia is favored if ≥55% of prolymphocytes are detected. However, the diagnostic process is intricate and relies on morphological criteria, as no reliable immunological or genetic marker has been identified. A substantial presence of circulating prolymphocytes indicates a potentially more aggressive form of CLL [55].

CLL cells express the surface antigen CD5 in conjunction with B-cell antigens CD19, CD20, and CD23. Each clone of leukemia cells is restricted to the expression of either κ or λ immunoglobulin light chains. A recent standardization effort has affirmed that a panel consisting of CD19, CD5, CD20, CD23, κ, and λ is typically adequate for establishing the diagnosis. In ambiguous cases, markers such as CD43, CD79b, CD81, CD200, CD10, or ROR1 may be useful in refining the diagnosis [56].

The immunophenotype of CLL cells has been incorporated into a scoring system designed to aid in distinguishing between CLL and other B-cell leukemias during the differential diagnosis. The categorization of leukemic mature lymphoproliferative disorders (LPD) through flow cytometry has traditionally revolved around the Moreau score, which was introduced in 1997. The results of this study suggested that the SN8 antibody might be a useful marker to differentiate between CLL and non-CLL. The highest accuracy was found for SN8, followed by CD23 and CD5. Within the standard panel, CD5 and CD23, and to a lesser extent, CD22, emerged as the most reliably scored markers in CLL. However, these markers may also yield positive scores in non-CLL cases. Conversely, SmIg and, to a lesser degree, FMC7 stand out as markers with the lowest occurrence of false-positive scores in non-CLL scenarios [55].

In a recent study involving patients with LPD, it was observed that a significant proportion of individuals with LPD received different classifications in flow cytometry depending on the scores or diagnostic systems utilized. The analysis encompassed all published scores and diagnostic systems for CLL, revealing suboptimal concordance among them. Consequently, it concluded that relying on score-based flow cytometry assessment for LPD may not be ideal, especially in the current landscape where multiple scores are available without a consensus on their use or performance. Despite the substantial overlap in the markers considered and how they are evaluated, the study results imply that flow cytometric classification is somewhat variable, often hinging on dichotomous determinations of continuous variables. The findings suggest that, if employed, the most suitable scores or diagnostic systems for each flow cytometry unit are likely influenced by various technical factors (such as the availability of antibodies) and interpretive preferences (e.g., fluorescence intensity versus the percentage of positive cells). Exploring non-score-based systems, such as the “full phenotype” system, appears to be a worthwhile consideration. Finally, the study underscores the importance of evaluating the reproducibility of the integrated diagnosis of leukemic LPD, encompassing not only flow cytometry but also cytology, cytogenetics, and molecular biology [57].

Numerous additional tests, although not mandatory for confirming a CLL diagnosis, are essential in assessing the patient’s prognosis and clinical condition. Table 3 delineates the diagnostic factors specified in different guidelines for diagnosing CLL.

**Figure 2:**
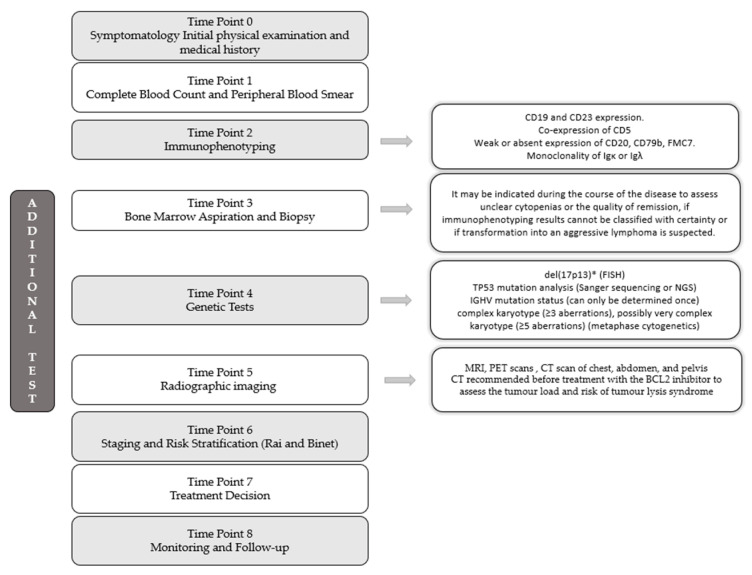
A time-dependent evaluation of diagnosis.

The diagnostic procedure depends on the primary set of findings, usually characterized by the key finding of lymphocytosis with or without accompanying lymphadenopathy. A time-dependent evaluation is shown in Figure 2.

There are two widely accepted systems for use in both clinical practice and clinical trials: the Rai [61] and Binet [62] classifications. These two staging systems are simple, inexpensive, and based on standard physical examinations and laboratory tests. They do not require imaging techniques without considering imaging techniques. They provide information on tumor burden and the prognosis of patients. However, these scales do not identify patients with aggressive behavior, especially in the early stages, nor do they identify the possible response to a given treatment.

The Rai staging system categorizes low-risk disease as individuals with lymphocytosis and the presence of leukemic cells in the blood and/or marrow (lymphoid cells > 30%)—formerly classified as Rai stage 0. Intermediate-risk disease, previously designated as stage I or stage II, is characterized by lymphocytosis, enlarged lymph nodes in any location, and the presence of splenomegaly and/or hepatomegaly. Patients with these features who have anemia (hemoglobin (Hb) less than 11 g/dL) (formerly stage III) or thrombocytopenia (platelet count less than 100 × 10^9^/L) (formerly stage IV) are considered high-risk disease patients [11,61].

Moreover, the Binet staging system relies on the enumeration of affected regions, characterized by the presence of enlarged lymph nodes exceeding 1 cm in diameter or organomegaly, along with the existence of anemia or thrombocytopenia. The implicated areas encompass (1) the head and neck, including Waldeyer’s ring; (2) axillae; (3) groin, including superficial femoral; (4) a palpable spleen; and (5) a palpable (clinically enlarged) liver. Within the Binet staging system, stage A is defined by Hb ≥ 10 g/dL and platelets ≥ 100 × 10^9^/L, with involvement of up to two of the aforementioned regions; stage B includes Hb ≥ 10 g/dL and platelets ≥ 100 × 10^9^/L, with organomegaly exceeding that defined for stage A; and stage C is characterized by Hb less than 10 g/dL and/or a platelet count less than 100 × 10^9^/L [62].

Due to recent advances in CLL treatment, these clinical staging systems have become insufficient to distinguish prognostic subgroups as they do not consider the aberrations in genetics or chromosomes discussed previously [11]. Consequently, the CLL international prognostic index (CLL-IPI) [63] was developed that combines clinical, biological, and genetic information [11,64,65,66]. This system employs five prognostic factors: TP53 deletion and/or mutation, variable immunoglobulin heavy chain mutational status, serum β2-microglobulin, clinical stage, and age.

According to the prognostic factor score, the patients will be classified as a risk group (low, intermediate, high, and very high risk) associated with a 5-year overall survival (OS) [63]. Its main limitation is that it has been validated in patients treated mainly with immunochemotherapy, its applicability has not yet been clearly demonstrated for new treatments directed at specific targets, and it can only be used in the prognostic evaluation of patients if the necessary molecular studies are available. Therefore, it is not a score that is used in clinical practice [30].

Despite the progress made with the implementation of the CLL-IPI index, factors such as tumor metabolism during leukemogenesis and the importance of nutritional status remain underrepresented [67]. Hypocholesterolemia has been reported in oncohematological disorders. Decreased levels of total cholesterol, HDL-C, and LDL-C were observed in patients with multiple myeloma [68], lymphoma [69], and, most recently, in patients with newly diagnosed CLL [70]. One study [67] developed a prognostic nomogram including these analytical parameters at the time of diagnosis as a significant predictive predictor. However, there is not enough evidence to incorporate it into clinical practice. 

CLL has one of the strongest hereditary predispositions for hematologic malignancies. As many as 10% of individuals who develop the disease have a prior family history. Other risk factors that have been found are living on a farm or exposure to herbicides and pesticides, a medical history of atopic conditions, exposure to hepatitis C, and common infections [71].

Further mutations or chromosomal changes acquired throughout the course of the disease contribute to a more aggressive pathology that becomes resistant to treatment. Deletion of chromosome 13q del(13q) is observed in around 55% of cases, while the acquisition of chromosome 12 (trisomy 12) occurs in 10–20% of cases. Del(11q) is present in roughly 10% of cases and del(17p) in approximately 5–8% of cases, although these aberrations typically occur in the later stages of the disease [72].

Genetic mutations influence disease prognosis and response to treatment. Therefore, patients with mutated VIGCs are associated with a better prognosis [73]. Recurrent mutations in MYD88 have been tentatively linked to a positive prognosis, although the evidence is not definitive [40]. Conversely, individuals with del(17p) exhibited poorer survival, whereas those with del(13q) or trisomy 12 demonstrated more favorable outcomes. Patients harboring del(11q) displayed an intermediate survival rate. CLL patients with mutations in TP53, NOTCH1, SF3B1, ATM, or BIRC3 were associated with an unfavorable prognosis [28,30,33,34,35,36].

The prognostic and therapeutic implications of TP53mut alterations are noteworthy. Its presence, as well as that of del(17p), implies an adverse prognosis and a poor response to conventional CIT. The PFS and OS of patients with del(17p) and those with TP53mut in the absence of del(17p) are similar, and therefore it is mandatory to study both in patients requiring treatment, both first-line and relapsed. It is also relevant that the incidence of TP53 alterations increases progressively as the disease progresses. This phenomenon is mainly because chemotherapy-resistant subclones are not eliminated and progress years later. Patients who received FCR or BR have much higher rates of TP53mut at relapse than at baseline [74]. This increase is also observed in other alterations with a poor prognosis, such as NOTCH1, SF3B1, and BIRC3, although not as markedly [32,75].

Genomic aberrations at CLL diagnosis, such as TP53 disruption, trisomy 12, and NOTCH1 mutation, increase the risk of Richter transformation (RT). RT is identified by a shift in histopathology and biology from the original CLL. It is characterized as the emergence of an aggressive lymphoma in individuals previously diagnosed with or concurrently experiencing CLL [76]. Additional risk factors contributing to the development of RT include bulky lymphadenopathy or hepato-splenomegaly, elevated beta-2-microglobulin, low platelet count, advanced disease stage, prior CLL therapy involving a combination of purine analogs and alkylating agents, and a higher number of lines of therapy [76,77].

It is linked to an unfavorable prognosis, with a median survival of less than one year. Managing the condition is intricate, and currently, available therapeutic approaches yield limited success in achieving sustained responses. Initial treatment for RT typically involves anthracycline-based chemotherapy regimens like R-CHOP (rituximab, cyclophosphamide, doxorubicin, vincristine, and prednisone) or R-EPOCH (rituximab, etoposide, prednisolone, vincristine, cyclophosphamide, and doxorubicin), as well as platinum-containing regimens such as ESHAP (etoposide, methylprednisolone, cytarabine, and cisplatin) and DHAP (dexamethasone, cytarabine, and cisplatin). Over the last five years, several new therapeutic options have emerged as possible treatments for individuals with B-cell malignancies, and their effectiveness in managing patients with RT has been assessed. These encompass targeted small-molecule inhibitors, innovative monoclonal antibodies (mAb), and approaches centered on stimulating an anti-tumor immune response, notably chimeric antigen receptor (CAR-T) cell therapy and T-cell-engaging bispecific antibodies [78].

## 5. Selecting the Right Treatment: How to Treat CLL?

Currently, initiation of treatment in patients with CLL in the first-line and relapsed disease should only consider patients with active or symptomatic disease. Initiation of treatment is recommended if the patient meets one of the criteria described in iwCLL guidelines [5].

Asymptomatic patients with early-stage CLL (Rai 0, Binet A) have not demonstrated the benefit of early therapeutic interventions. Therefore, they are initially managed without pharmacological treatment.

When initiating treatment for CLL, the patient’s age, the presence of alterations in renal, cardiac, pulmonary, hepatic, and immunological functions, and life expectancy should be considered. The Cumulative Illness Rating Scale (CIRS) is the most widely used in the different clinical trials of CLL when measuring comorbidities [79].

Although CLL predominantly affects older adults, risk stratification systems for CLL have not focused on geriatric domains, such as subjective and objective measures of function and cognition [80]. This patient profile is characterized by medical and psychosocial problems that affect their ability to tolerate treatment and contribute to negative outcomes and increased morbidity. The unfit patient is, therefore, defined as that fraction of patients with marked comorbidity that prevents them from tolerating CIT regimens [17]. Thus, a comprehensive geriatric assessment (CGA) can help to robustly characterize health status and represent a better measure of the health of elderly patients than a simple assessment of functional status or consideration of chronological age alone.

Although most studies have been reported in patients with solid tumors [81,82], studies demonstrate the importance of geriatric assessment in hematological malignancies [83,84,85,86]. Previous research on older adults undergoing chemotherapy for various hematologic malignancies has identified a correlation between the degree of geriatric impairments and OS. [80,85,86,87,88]. 

In “unfit” patients, in parallel to assessing the presence and type of comorbidities and the existence of concomitant treatments, it is important to evaluate the convenience of prescribing indefinite or time-limited treatment and the convenience of indefinite versus time-limited treatment. A finite treatment has the advantage of higher patient convenience, and it is usually associated with lower toxicity [17]. It is important to evaluate not only the treatment’s effectiveness and safety but also drug accessibility, associated costs, and therapeutic objectives [89].

## 6. Treatment of CLL

### 6.1. Cytostatic Agents

Alkylating agents were the first therapeutic options for the treatment of CLL. Chlorambucil was the gold standard treatment until 1990 [90]. Despite the advantages of oral administration and its low cost, the overall response rate (ORR) oscillated between 35 and 65%. The limited efficacy combined with the side effects of prolonged use (cytopenia and myelodysplastic syndromes/acute leukemia) result in limited use of this treatment. A palliative prescription may be considered for elderly or unfit patients. The combination of chlorambucil with corticosteroids or other chemotherapy (CHOP) has not been shown to be superior to monotherapy [91].

Purine analogs were then introduced into CLL treatment, especially fludarabine, pentostatin, and cladribine. Fludarabine was notable for its superior ORR compared to other treatment regimens available at the time. However, fludarabine used as monotherapy did not demonstrate an increase in OS [92].

Bendamustine is an alkylating agent that is structurally intermediate between alkylating agents and purine analogs, with the advantage of lower hematological toxicity. It showed significantly superior efficacy to chlorambucil in the first line with significantly higher OR and PFS rates (29 vs. 4% and 68 vs. 39%, respectively). In relapsed or refractory (r/r) CLL, it demonstrated a superior OR rate to fludarabine (76 vs. 62%) and PFS (27 vs. 9%). The combination with mAb increases response rates [93].

### 6.2. Monoclonal Antibodies

The predominant choices involve antibodies targeting CD20. It is believed that this protein functions as a calcium channel within the cell membrane. Given its presence in most B-cell malignancies, the incorporation of these antibodies has enhanced the treatment outcomes for CLL [94].

Rituximab was the first chimeric mAb directed against an epitope of this molecule that demonstrated an antitumor effect in virtually all mature B neoplasms. This agent can induce direct tumor lysis by apoptosis and activation of antibody-dependent cytotoxicity (ADCC) and antibody-dependent cytotoxicity (CDC) mechanisms. Its efficacy as a monotherapy agent in CLL or maintenance therapy is limited. The response rate is lower than other types of lymphomas, probably related to the lower expression density in CLL. However, its association with the chemotherapy regimens used (fludarabine, pentostatin, and cyclophosphamide) resulted in a significant improvement in the treatment of CLL [79].

Obinutuzumab is a humanized and glycoengineered mAb, which resulted in higher affinity binding to a type II CD20 epitope and greater direct induction of cell death. The GAUGUIN trial (phase 1/2) evaluated obinutuzumab monotherapy in patients with r/r CLL. It demonstrated that obinutuzumab was an active treatment. ORR was 62% (phase 1) and 30% (phase 2). Phase 2 median PFS was 10.7 months [95].

Ofatumumab is a humanized MoAct directed against a different CD20 epitope than rituximab, with a higher CDC lytic capacity and an ADCC similar to rituximab. In the randomized phase II trial of 201 patients with r/r CLL refractory to fludarabine and alemtuzumab or fludarabine/presence of mass greater than 5 cm, ofatunumab alone achieved an OR rate of 51% and 44%, respectively [96]. A clinical trial studied the efficacy and safety of ofatumumab versus ibrutinib. The OS rate was significantly higher for ibrutinib (hazard ratio for death in the ibrutinib group, 0.43; 95% CI, 0.24 to 0.79; *p* = 0.005), as was the response rate (42.6% vs. 4.1%, *p* <0.001). As a result, this treatment is rarely used in clinical practice [97].

Ublituximab is another anti-CD 20, a chimeric antibody with a higher affinity for FcγRIIIa/CD16 receptors and a higher ADCC compared to rituximab. As a monotherapy agent, it induces up to a 50% response in patients with r/r CLL. This agent is currently under development in combination with BTKi, PI3K, and B-cell lymphoma gene 2 (BCL-2) [98].

Alemtuzumab is an anti-CD52 antigen that is recombinant and fully humanized. In monotherapy, response rates range from 33% to 53%, and the median duration of response falls within the range of 8.7 to 15.4 months. This applies specifically to patients with advanced CLL who were previously treated with alkylating agents and experienced failure or relapse after undergoing second-line treatment with fludarabine. It is also a particularly active drug in patients with high-risk genetic markers such as del17p/TP53mut. The main limitation is its profound immunosuppression, with a high rate of opportunistic infections that require a triple antibiotic, antifungal, and antiviral prophylaxis and close patient monitoring. Its marketing license was removed in 2012, and it can only be used on a compassionate use basis [99,100].

Otlertuzumab is a single-chain anti-CD37 Ac capable of inducing a 23% response rate in monotherapy. It has also been combined with bendamustine and compared in a phase II trial to bendamustine alone, with a significantly higher response rate (69 vs. 39%; *p* = 0.02) and median PFS (15.9 vs. 10.1 months; *p* = 0.019) [101].

### 6.3. Chemoimmunotherapy

CIT remains a viable choice for fit patients with low- and intermediate-risk CLL. The combination of FCR stands out as a well-established standard of care for patients eligible for treatment without the presence of del(17p) and/or TP53mut. Several clinical studies have explored the application of CIT in CLL patients. Table 4 summarizes the main studies carried out on this subject.

It should be noted that CIT has a limited, if any, role in treating CLL because this class of drugs is inferior to BTKi and venetoclax-based regimens in different clinical settings. It also causes an increased risk of therapy-related myeloid malignancy and infections. In the CLL13 study trial, which was performed with venetoclax plus anti-CD20 antibodies as a first-line treatment in fit patients, it can be appreciated that treatment with VO or VO-ibrutinib produced a significant PFS benefit in IGHVum patients but not in IGHVm patients. In these patients, improving the high efficacy of the FCR regimen in young, generally well-conditioned patients may be difficult. However, as a finite therapy, venetoclax has the benefit of a lower risk of toxicity and adverse events (AE) associated with treatment, as well as second malignancies or clonal selection. Hence, the use of FCR is less and less recommended, even though numerous clinical guidelines still state the contrary [106].

### 6.4. Agents Targeting the Signaling in CLL Cells and in Their Microenvironment

A detailed study of the pathophysiology of the disease has been key to the design of specific molecules targeting several tyrosine kinases involved in the main intracellular signaling pathways that are key to tumor cell survival and proliferation, including BTK and PI3K.

#### 6.4.1. PI3K Inhibitors: Idelalisib, Duvelisib, and Umbralisib

Idelalisib is an irreversible inhibitor of the δ-subunit of the catalytic portion of PI3K, blocking the transmission of signals from BCR and reducing the phosphorylation of AKT. This results in reduced interaction with the cellular microenvironment. It is administered with rituximab. In the phase III trial comparing idelalisib + rituximab with placebo + rituximab, the response rate was 81 vs. 13% (*p* < 0.001), respectively, with a significantly higher OS at 12 months (92 vs. 80%; *p* = 0.02). This study showed that the drug was active in patients with 17p deletion and in patients with IGHVum. Drug toxicity severely limits its use, including high rates of myelosuppression, grade ≥ 3 transaminitis, and colitis [107].

Duvelisib is a dual PI3kδ and PI3Kγ inhibitor. The phase Ib monotherapy trial demonstrated 74% ORR in patients with r/r CLL. Although its activity is remarkable, and the FDA has approved it for treating r/r CLLL, its development has also been limited by toxicity (mainly hematological and hepatic toxicity) and has not been approved by the EMA [108]. In 2022, the FDA issued a safety alert warning about the possible increased risk of death and severe side effects such as infections, diarrhea, inflammation of the intestines and lungs, skin reactions, and elevated levels of liver enzymes in the blood. As a result, this treatment is not reflected in CLL clinical guidelines [109].

Umbralisib is a PI3Kδ inhibitor with a markedly different chemical structure from the ones above. The phase I trial showed 85% OR in patients with r/r CLL with a significantly lower frequency of hepatotoxicity and colitis [110]. Recently, the UNITY study explored umbralisib in combination with ublituximab in treatment-naïve and r/r CLL and provided a median PFS of 32 versus 18 months after a median follow-up of 36 months [111].

#### 6.4.2. BTK Inhibitors: Ibrutinib, Acalabrutinib, Zanubritinib, and Pirtobrutinib

##### Ibrutinib

This orally active, small-molecule BTKi triggers apoptosis in B-cell lymphomas and CLL cells. BTK participates in migration and tissue adhesion pathways. Its inhibition leads to a redistribution of neoplastic lymphocytes into the bloodstream, where they die via apoptosis. Patients generally respond to ibrutinib initially with a rapid reduction in lymph node size and transient peripheral blood lymphocytosis, which appears after 4–6 weeks and resolves spontaneously in 80% of cases within the first year of treatment [112]. Table 5 shows the main clinical studies carried out with this drug.

In addition, we found a phase II study providing information on the first-line use of ibrutinib in patients with CLL and del 17p/TP53 mut. In this study, 51 patients with CLL with del17p or TP53mut treated first-line or r/r were treated with ibrutinib. A response was achieved in 97% of first-line patients, and in r/r patients, 80% had a response. OS at 24 months was 80% in first-line patients and 74% in r/r patients [120].

The tolerability profile is very favorable; diarrhea, rash, muscle pain, spasms, and infections tend to progressively disappear after 6–12 months of treatment. Medium-term AEs include hypertension and complete atrial fibrillation arrhythmia. These side effects are the main cause of long-term drug discontinuation [121]. This treatment achieves one of the best remission durations documented to date. One of his main problems is that it is an indefinite therapy.

##### Acalabrutinib

It is a highly selective irreversible covalent inhibitor of second-generation BTK with lower affinity for IL-2-inducible kinase (ITK) and epidermal growth factor receptor (EGFR). This lower affinity theoretically results in a reduction of the side effects of ibrutinib. It is indicated as monotherapy or in combination with obinutuzumab for treating adult patients with CLL who are previously untreated or have received at least one previous treatment [122]. Table 6 summarizes the clinical trials performed with acalabrutinib.

##### Zanubrutinib

It is a second-generation oral BTKi that irreversibly and covalently binds to the catalytic region of BTK, blocking its function. It proved to be more selective than ibrutinib against BTK in relation to other similar enzymes. Particularly remarkable is the selectivity of zanubrutinib for ITK and EGFR enzymes, resulting in less inhibition of T and NK cell function and greater antibody-dependent cytotoxicity for zanubrutinib [128].

However, for HER4, the difference between zanubrutinib and ibrutinib was marginal, and there were no significant differences with respect to three other kinases: BMX/ETK, BLK, and TXK [128]. As we can see in Table 7, there are several studies carried out with zanubrutinib.

The most frequently observed AEs included infections, neutropenia, and diarrhea. In general, events leading to treatment discontinuation were less prevalent with zanubrutinib compared to ibrutinib [120,130].

##### Pirtobrutinib

It is a highly selective yet reversible BTKi, exhibiting efficacy in patients with the C481S mutation of BTK.

BRUIN TRIAL: Open-label phase I/II trial. This study focused on determining the maximum tolerated dose (phase I) and ORR (phase II) of pirtobrutinib. The trial included patients treated for CLL or SLL. Pirtobrutinib exhibited an ORR of 62% in these individuals. ORR remained consistent across different CLL subgroups, including those with previous covalent BTKi resistance (67%), covalent BTKi intolerance (52%), C481-mutant BTK disease (71%), and wild-type BTK (66%). These results suggest that reversible BTKi, such as pirtobrutinib, may provide an alternative for patients facing intolerance or resistance to traditional BTKi [131].

### 6.5. BCL-2 Inhibitors

Venetoclax: It is a BCL-2 inhibitor (BCL-2i) that binds directly to the BH3 binding site. It displaces proapoptotic proteins with BH3 domains and initiates mitochondrial outer membrane permeabilization, activating the caspase pathway and programmed cell death. Thus, venetoclax depletes dendritic cells and total lymphocytes while reducing interferon α production [132]. Table 8 lists the main studies with venetoclax alone or in combination with other drugs.

### 6.6. Lenalidomide

It is a 4-aminoglutamyl analog of thalidomide with activity against various hematological malignancies. It has been shown to be an immunomodulator that affects the immune system and has anti-angiogenic properties. In people with CLL, lenalidomide might interact with cancer cells, affecting how CLL cells and their surroundings interact. The effectiveness and safety of using lenalidomide as an ongoing treatment for CLL are still uncertain and debated in different studies [140]. The ORR of lenalidomide monotherapy ranged from 32% to 54% [141]. In a long-term study of 60 patients, an OS of 82% was observed. Thirty-five (58%) patients had a response lasting longer than 36 months (long-term responders [LTR]). The best long-term responses were 25 (71%) CR and 10 (29%) PR [142]. Other studies of lenalidomide in maintenance versus placebo after second-line therapy did not demonstrate a significant improvement in OS [143].

### 6.7. Other Therapies: Allogeneic Transplantation and CAR-T

New targeted therapies have led to a decline in the utilization of allogeneic hematopoietic stem cell transplantation (alloHCT) in CLL patients. This shift is attributed to the significant morbidity risk associated with alloHCT, which includes organ toxicity, as well as the potential for acute and chronic graft-versus-host disease. Nevertheless, this therapy still has an important role, particularly for eligible patients with high-risk genetics. Within the category of high-risk patients, two distinct groups can be identified: high-risk I, characterized by clinically resistant disease to CIT with TP53 aberrations but a positive response to signaling pathway inhibitors, and high-risk II, marked by disease resistance to both CIT and signaling pathway inhibitors. In cases where resistance to BTKi and/or BCL-2 is observed, alloHCT could be a viable alternative, especially when therapeutic options are limited [144].

When choosing a patient for alloHCT, factors such as the patient’s functional status, age, comorbidities, donor availability, status of del17p and TP53mut, prior treatment history and response duration, the level of response to the current therapy, and the availability of alternative treatment options should also be considered [145]. The benefits of alloHCT in CLL have never been confirmed with a randomized controlled trial; large data sets from retrospective studies demonstrate that alloHCT achieves durable remissions in up to 30–50% of patients with heavily pretreated CLL [146,147]. There is a suggestion that alloHCT is linked to a reduced risk of relapse and enhanced survival. However, it is crucial to interpret these findings with caution as the studies were conducted before the emergence of BTKi and BCL-2i, and there is a potential risk of selection bias [148].

Another therapy that is under study for refractory patients is CAR-T. In one study, 24 patients diagnosed with CLL who had previously undergone ibrutinib treatment were administered anti-CD19 CAR-T cell therapy. Four weeks following the infusion of CAR-T cells, the ORR stood at 71% (17 out of 24). Among the 19 patients re-evaluated, the ORR four weeks post-infusion was 74% (CR, in 4/19, 21%; Partial Response (PR) in 10/19, 53%). Moreover, 88% of the patients (15 out of 17) who had marrow disease before CAR-T cell treatment exhibited no disease via flow cytometry after CAR-T cells, and in 7 patients (58%), no malignant IGH sequences were detected [149].

In the TRANSCEND-CLL004 study, individuals with r/r CLL received lisocel as monotherapy, delivered in equal proportions of CD8+ and CD4+ CAR-T cells (23 patients), or a combination of lyocell and ibrutinib to enhance engraftment by BTKi (19 patients). Both groups displayed an ORR exceeding 90%. Cytokine release syndrome occurred in 74% of patients (9% graded as 3), and neurological events were observed in 39% (22% graded as 3/4) [150].

## 7. Selection of First-Line Treatment of Symptomatic Patients according to Clinical Guidelines and Expert Consensus

### 7.1. Treatment of Patients with del(17p) and/or TP53 Mutation

Aberrations in TP53 have been acknowledged to impart an unfavorable prognosis concerning response rate, PFS, and OS, especially with CIT but also with novel agents. However, there has not been a randomized clinical trial specifically exploring patients exclusively with del(17p) and/or TP53-mutated CLL. There are several recommendations regarding the treatment of patients with del(17p) or TP53mut. They are summarized in Table 9.

### 7.2. Treatment of Patients with No TP53 Aberrations or del 17p

Patients ought to be categorized based on the mutational status of the IGHV gene locus, distinguishing between mutated and unmutated variants. In instances where prospective stratification proves challenging, the analysis should involve a subgroup assessment of IGHVm and IGHVum patients.

#### 7.2.1. Mutated IGHV

As shown in Table 10, the recommendations made by the different clinical guidelines are very similar, distinguishing between FIT and non-FIT patients and with differences in their therapeutic approach, which we will see below.

#### 7.2.2. Unmutated IGHV

Patients with the IGHVum gene have an inferior outcome to those with the mutated IGHV gene. Table 11 shows the recommendations according to the different clinical practice guidelines for the treatment of this group of patients.

According to JAMA, first-line treatment in patients with normal TP53, regardless of IGHV status, consists of either VO (a fixed-duration treatment) or covalent BTKi such as acalabrutinib, Zanubrutinib, or ibrutinib. Second-generation BTKi (acalabrutinib and zanubrutinib) is preferred, given improved safety. Also, zanubrutinib had superior efficacy compared to ibrutinib [154].

## 8. Rescue Treatment in Relapsed/Refractory Patients

The understanding of novel agent therapy is advancing with the progression of clinical trial data. This stems from the fact that a significant proportion of patients enrolled in registration trials that led to the approval of novel agents did not undergo prior novel agent treatments; however, robust retrospective analyses have provided significant guidance [157]. Individuals resistant to current therapies or those experiencing remission periods of less than 2 to 3 years, along with patients who relapse and exhibit evidence of del17p or TP53mut, face an unfavorable prognosis. Prior to selecting further treatment for any patient with r/r CLL, it is crucial to evaluate whether the treatment criteria outlined in the iwCLL 2018 guidelines are met [13].

Performing FISH to detect del17p and conducting tests for TP53mut is recommended before initiating the first-line treatment and at each subsequent treatment stage for r/r CLL patients. The mutational status of IGHV does not change during the disease, and repeat testing is not indicated. In the realm of r/r CLL patients, innovative treatments outperform conventional CIT regimens, resulting in markedly improved survival rates. The selection of therapeutic agents is contingent upon diverse factors, including age, functional capacity, comorbidities, organ functionality, and patient preferences [1]. Some factors have been identified as prognostic in CLL, at both treatment-free interval and OS level. Certain factors contributing to this include the presence of an IGHVum gene, cytogenetic abnormalities like a complex karyotype, del(17p), TP53mut, and others. The temporal aspect of disease progression also stands as a crucial prognostic element in relapsed CLL patients [15].

The early onset of disease progression, defined as occurring within 24 months of frontline therapy, is a recognized predictor of lower response rates to subsequent therapy and diminished survival. The context of relapse is also noteworthy, with disease recurrence following finite-limited therapy potentially treated using the same regimen, provided the initial therapy’s response duration is satisfactory [158]. Patients experiencing disease progression after maintaining a response for at least 6 months are categorized as having relapsed CLL. On the other hand, those who show no response to treatment or relapse within 6 months of the last therapy dose are classified as having refractory CLL. The subsequent approach for both r/r cases depends on their prior treatment histories.

According to the recommendations of the SGCCL [151], iwCLL/NCNN [1,156], and DGHO [155] guidelines, treatment for those patients who have undergone prior CIT should be acalabrutinib, zanubrutinib, ibrutinib (only in cases where acalabrutinib and zanubrutinib are contraindicated), or VR. The SGCCL guideline [151] distinguishes between TP53mut/non-del(17p) patients (within these, those with IGHVm or IGHVum) and patients with TP53mut/del(17p). In all cases, treatment is as previously recommended according to the patient profile. DGHO guidelines [155] recommend stopping therapy after 24 months of treatment with late-relapsed VR.

On the other hand, those patients who have received a previous BTKi and have not responded to it should be treated with others such as acalabrutinib or zanubrutinib (in case the patient does not present resistance to BTKi), VO for 24 months [1,156], or VR (stop after 24 months of treatment) [151,155]. Those patients experiencing intolerance or early relapse (less than 24–36 months of treatment) or those patients with late relapse who have del(17p)/TP53mut and have been treated with a prior BCL-2i are recommended to be treated with acalabrutinib, Zanubrutinib, or in those who do not respond to these treatments, clinical trials, or cell therapy [151]. Ibrutinib is recommended when acalabrutinib and zanubrutinib are contraindicated [155], and VO can be used (for 24 months) [1,156]. Patients with non-del(17p)/TP53mut late relapse who have IGHVm or IGHVum can be treated with either VR, acalabrutinib, Zanubrutinib, or ibrutinib according to clinical practice guidelines.

If the patient has been doubly refractory (which means not responding to BCL-2i and BTKi), guidelines recommend including the patient in clinical trials and using cell therapy (CAR-T) or idR in exceptional cases according to the safety profile. For frail patients, supportive care is the preferred option [151,155,156].

JAMA guidelines consider that if the patient is intolerant or the disease progresses to first-line treatment, there are several options. Patients previously treated with BTKi who are intolerant to the treatment can be treated with other BTKi or with VR (in those with rapidly progressive disease, inpatient care with rapid dose escalation should be considered). If the patient is treated with a BTKi and experiments with disease progression after it, the treatment should be VR (considering rapid dose escalation in those with rapidly progressive disease) or a non-covalent BTKi inhibitor such as pirtobrutinib (when available) [154].

Patients previously treated with venetoclax who experienced progression while receiving treatment or early after discontinuation can be treated with acalabrutinib, Zanubrutinib, or ibrutinib (second-generation BTKi are preferred, given improved safety based on clinical trials). Also, non-covalent BTKi inhibitors such as pirtobrutinib can be used when available. If the patient experiences a late progression after discontinuation, they can be treated with acalabrutinib, zanubrutinib, ibrutinib, or pirtobrutinib, and retreatment with venetoclax can be considered [154,159].

Finally, if the disease progresses after BTKi or venetoclax, either noncovalent BTKi (pirtobrutinib) or PIK3 inhibitors (idR or durvalumab) can be used. Pirtobrutinib is preferred over PIK3 inhibitors, as its efficacy has been shown in a clinical trial, including after receipt of BTKi and venetoclax. At least, cellular immunotherapy should be considered. CAR-T therapy is recommended when available in patients with controlled disease (while responsive to treatment) and alloHCT if there is no access to CAR-T or after CAR-T [154].

ESMO guidelines consider that first-line therapy should only be used in symptomatic patients. Patients with relapsed and asymptomatic CLL can be followed without therapy. If CLL is in remission, stopping BTKi or venetoclax can be considered and does not need an immediate alternative. If CLL progresses rapidly, therapy should be changed immediately. If a symptomatic relapse or non-response appears within 3 years after a fixed-duration therapy regimen, it should be changed regardless of the type or first-line treatment (contrary to the most recent guidelines that consider previous treatments) [58].

Treatment should be either VR for 24 months, ibrutinib/acalabrutinib, or other BTKis (if available) as continuous therapy. Alternative options include PIK3 inhibitors such as idelalisib in combination with rituximab or CIT unless a TP53 mutation or del(17p) is found and no other treatment options with inhibitors or cellular therapy are available, and it is not recommended because it increases toxicity rates and the risk of secondary neoplasm. When a progression is observed on BCR inhibitor (BCRi) therapy after prior CIT, venetoclax-based therapy is preferred because it has been observed that changing to a different CIT or BCRi does not induce long-lasting remissions. Patients may be re-exposed to the same treatment regimen when there is a long-lasting remission (more than 36 months) from prior therapy [58].

There are several studies, such as CLARITY [160], in which a phase II trial was conducted, investigating the combination of ibrutinib and venetoclax in individuals with r/r CLL. The primary objective was the elimination of MRD after 12 months of concurrent therapy. Key secondary objectives included evaluating responses according to iwCLL criteria, ensuring safety, and assessing PFS and OS. The combination of ibrutinib and venetoclax demonstrated good tolerability in patients with r/r CLL. A notable proportion of patients achieved MRD eradication, leading to the discontinuation of therapy in some cases.

The PFS and OS rates were encouraging for individuals with r/r CLL [161]. Venetoclax response rates ranging from 71% to 79% have been observed among patients in subgroups with an adverse prognosis, such as those with fludarabine resistance, del(17p), or IGHVum. The 15-month PFS for the 400 mg dose groups was 69%. In another trial in patients with r/r del(17p) CLL, an OR was achieved by independent review in 85 patients (79.4%) [162].

There is a lack of data from randomized clinical trials directly comparing novel agents. Nevertheless, indications propose that individuals experiencing late relapse (beyond 2 years) following fixed-duration therapies may derive benefits from identical retreatment. In contrast, those with short-lived remissions or progressive disease under continuous drug intake may find a favorable outcome with a class switch. The treatment of patients previously exposed to both covalent BTKi and BCL-2 remains an unresolved medical challenge. Early clinical trials indicate the promising efficacy of novel drugs, especially noncovalent BTKi, in addressing the therapeutic needs of this challenging subgroup [163].

## 9. Treatment Resistance

The emergence of resistance to treatment is related to the progression of subclones with clear proliferative advantages. Subclones that are not eliminated via chemotherapy can lead to disease progression years later. Therefore, in patients treated with CIT, the acquisition of TP53 mutations or deletions is not uncommon [74].

On the other hand, the acquisition of mutations at the BTK or BCL-2 level is a cause of resistance to ibrutinib and venetoclax, respectively. Concerning BTKi, the mechanism of resistance is similar among the covalent BTK inhibitors. Switching between drugs in this category after disease progression should be avoided. When treatment is deemed necessary, clinical trials have demonstrated the effective use of acalabrutinib or zanubrutinib in patients intolerant of ibrutinib. Additionally, zanubrutinib can be employed in patients intolerant of either ibrutinib or acalabrutinib. The preference for zanubrutinib and acalabrutinib over ibrutinib is supported by their favorable safety profiles, and zanubrutinib exhibits superior efficacy compared to ibrutinib. Findings from the E1912 study indicate that the median time between discontinuing ibrutinib due to AE and initiating new therapy is 25 months [160].

In very pre-treated patients, acquired resistance to ibrutinib can emerge, primarily mediated through the mutation of BTK cysteine-481—the amino acid that ibrutinib irreversibly reacts with—to serine.

This mutation blocks the covalent binding of ibrutinib, resulting in the inability of ibrutinib to exert its therapeutic effect. This same resistance pattern has also been observed with acalabrutinib and zanubrutinib, although the incidence of resistance associated with these drugs requires further investigation [164]. Non-covalent BTK inhibitors were developed to improve pharmacological properties and maintain potency against BTK C481 mutations. However, BTK L528W-mediated resistance mechanisms have recently been observed for pirtobrutinib [165]. BTK L528W also leads to a decrease in the pharmacological potency of zanubrutinib [166].

Progression to ibrutinib treatment has also been observed in patients with del 8p. The presence of mutations in PLCG2 (R665WW and L845F mutations) leads to autonomous BCR activity [167]. The BCL-2 G101V mutation diminishes the binding strength of venetoclax to BCL-2 by 180-fold. This selective reduction in affinity contributes to resistance to therapy [168].

## 10. Where Are We Going in the Therapeutic Approach to CLL?

Selecting therapy is a challenging task that requires evaluating the patient regarding all aspects that could interfere, such as other diseases and patient preferences. Also, side effects, comedications, and economic aspects should be considered. Updated NCCN guidelines and the recent iwCLL algorithm recommend second-generation BTKis, such as zanubrutinib and acalabrutinib, for the treatment of naïve patients and r/r CLL regardless of patient fitness due to their increased selectivity and favorable drug toxicity profiles [156].

Fixed-duration chemo-free therapies in CLL, which can induce a complete response, are becoming more important in treating CLL. The CIT approach has a marginal role in r/r CLL nowadays, and therapies such as venetoclax and antiCD20 mAbs are alternatives that must be discussed in each patient, considering advantages and disadvantages. Offering the best treatment option is a challenge nowadays and requires a deep knowledge of the growing evidence [169].

Some studies show that despite advances in CLL treatment regarding efficacy and tolerability, premature treatment discontinuation is frequent in all types of therapies. The feasibility of the fixed-duration treatment regimen is nowadays a question unsolved. Targeted therapies have typically been used continuously until disease progression. This poses problems for patients, such as economic expense or intolerance to the drug, which can lead to dose reductions or even a lack of treatment adherence, sometimes leading to treatment abandonment. Patients with increasing comorbidities (advanced age or health problems) may be especially prone to lower tolerability of long-term drugs, so fixed-duration treatment may be a very good option for them. This can be seen in the MURANO [135] study, where it was seen that 70% of patients maintained the achieved level of uMRD, and among patients who achieved uMRD, 98% of patients did not progress. It is important for prescribers to consider the problem of sustainability. That is why the option of using fixed-duration treatments represents an opportunity to provide access to treatments that are sustainable for the national health system, and in cases where there is no evidence of benefits from a certain treatment over another potentially equally effective and tolerable one, it could help the prescribers as an important factor to consider. Additional studies are needed to evaluate the efficacy of these fixed-duration therapies and the impact of treatment discontinuation rates [170].

Ongoing investigations into triple therapy are yielding exceptionally promising outcomes. Time-limited triple therapy demonstrates elevated rates of achieving undetectable minimal residual disease and maintaining remissions in individuals with high-risk CLL. The occurrence of AE is early in the induction therapy phase and diminishes as treatment progresses. The CLL2-GIVe study assesses the response and tolerability of the triple combination comprising obinutuzumab, ibrutinib, and venetoclax (GIVe regimen) in forty-one previously untreated high-risk CLL patients with del(17p) and/or TP53 mutation. The 36-month PFS was 79.9%, and the 36-month OS was 92.6% [138]. Time-limited triple therapy with obinutuzumab, venetoclax, and acalabrutinib was evaluated in 37 patients with previously untreated CLL. The primary endpoint was complete remission with uMRD in the bone marrow at cycle 16, day 1. The results showed that 86% of participants had a complete remission with uMRD in the peripheral blood and bone marrow [171]. In a trial with zanubrutinib, venetoclax, and obinutuzumab in 39 previously untreated CLL patients, 89% of them had undetectable MRD in both blood and bone marrow (median follow-up 25.8 months, IQR 24.0–27.3). After median surveillance after treatment of 15.8 months (IQR 13.0–18.6), 31 (94%) of 33 patients had undetectable MRD [172]. The existing data on targeted therapies face several limitations. Firstly, the comparison between triplet combinations with sequential single novel agent therapies is lacking. Although combination regimens have generally been well-tolerated, they do exhibit a level of toxicity higher than that of single agents. Therefore, it is imperative to identify those who benefit most from combination therapy and those who would be better served by less toxic sequential monotherapies. When considering doublet versus triplet regimens, it is essential to acknowledge that the individual contributions of drugs in combination regimens, especially CD20 mAbs, remain unclear [173].

## 11. Take-Home Message

What is success in CLL? The goal in CLL, as in any cancer, remains to prolong the quality of life and overall survival while offering the least possible toxicity. To achieve this, it is necessary to specify what we bring to the table in terms of efficacy and safety with combination treatments. While the current data suggest that the combinations have a present and a bright future, it is necessary to do so from the point of view of the heterogeneity of CLL patients, taking into account the age, the life perspectives of the patients, and the fact that it is a chronic and incurable disease, resulting in the treatment target and the toxicities to be assumed being very different. Therefore, knowing the chronic nature of hemopathy, treatment must be individualized according to three fundamental pillars (or three vertices of a triangle): the patient (age, frailty, comorbidities, concomitant medication, hospital accessibility, and preferences), the disease (genetic status, risk markers, acquired resistances, and expected efficacy obtained with the treatment), and the physician (accessibility and approval of treatments in your environment, experience with treatments, training,…). We must also bear in mind that strategies guided by minimal residual disease are a vital part of today’s CLL clinical trials, although we need to know what impact they really have on different patients since the treatment objective is not the same in someone aged 77 who is going to relapse at 83 as in someone we are treating at 40, and yet we do know that the toxicity of continuing indefinite treatments increases over time. It is clear that the paradigm of CLL treatment is changing; we have gone from treating patients with aggressive chemotherapy to being able to predict a targeted treatment with less toxicity, which is even giving us the option of being able to stop treatment in certain patients with what this entails in terms of improved quality of life, improved adherence to treatment, less toxicity in the long term, greater sustainability of the health system, and better health outcomes. All this must be carried out through a multidisciplinary approach (hematologist, hospital pharmacist, and nursing) in order to offer personalized and specialized care and to be able to offer the patient adequate and individualized treatment.

## 12. Conclusions

Thanks to new therapeutic targets, the current panorama is moving towards more sustainable, personalized treatments with less risk of toxicity for patients, trying to adapt the therapy to the profile of the patient to whom it is directed. This requires the help of a multidisciplinary team working together to achieve the best outcome for the patient and the health system. The increased progressive knowledge of the disease through genetic techniques and its consequent therapeutic implications allow us to better predict the disease, anticipate diseases of more or less risk, detect resistance mutations, or follow up on minimal residual disease. The development of new treatment strategies, such as fixed-duration chemo-free and time-limited triple therapy, represents a new paradigm in the treatment of the disease, offering benefits at the level of acquired resistance, costs, accumulated adverse effects, and probably at the emotional level (offering treatment-free and disease-free periods), so we believe that in the coming years, they will oppose continuous therapies until disease progression.

## Figures and Tables

**Table 1:** Mutations implicated in CLL.

Mutation	Prevalence	Location	Signaling Pathway	Prognosis	References
TP53	5–10% at the beginning of treatment 40–50% in refractory patients	Chromosome 17	Resistance to apoptosis induced via DNA-damaging agents	Very poor prognosis	[17,18]
BIRC3	2–6%	Chromosome 11	NF-κB signaling	Poor prognosis	[26,27,28]
NOTCH1	10–15%	Chromosome 9	NF-κB signaling	Poor prognosis	[29,30,31]
ATM	10–12%	Chromosome 11	Aberrations in DNA repair mechanisms	Poor prognosis	[32,33,34]
SF3B1	5–10%	Chromosome 2	Splicing RNAm	Poor prognosis	[35,36]
MYD88	3%	Chromosome 3	NF-κB signaling	Good prognosis	[36,37]

**Table 2:** Role of cytokines in CLL.

Cytokine	Action	Mechanism	Ref.
Il-2	Improves the function of CLL cells.	Promotes the differentiation and proliferation of CLL cells.	[41]
Il-4	Increases CLL cell survival and proliferation. Reduces apoptosis.	Activates JAK/STAT signaling pathway and enhances expression of antiapoptotic proteins.	[42]
Il- 6	Increases CLL cell survival and proliferation.	Activates JAK/STAT signaling pathway and enhances expression of antiapoptotic proteins.	[43]
Il- 8	Survival and chemoresistance of CLL cells.	Activates JAK/STAT signaling pathway.	[44]
Il-9	Stimulates growth and survival of CLL cells.	Activates the JAK/STAT pathway and the phosphatidylinositol phosphokinase subunit 3 (PI3K)/Akt/mTOR signaling pathway.	[45]
Il- 10	Suppresses anti-tumor immunity.	Reduce the production of effector CD4 and CD8 T cells.	[46]
Il- 17	Increases CLL cell survival and proliferation.	Activates the NF-κB pathway and increases the expression of antiapoptotic proteins.	[47]
TNF-α	Induces apoptosis.	Attaches to the TNF receptor found on CLL cells, activating caspases to induce programmed cell death.	[48]
IFN-γ	Inhibits proliferation.	Attaches to the IFN-γ receptor present on CLL cells, initiating the JAK-STAT pathway to hinder cell proliferation.	[49]

**Table 3:** The amendments from iwCLL, ERIC, and ESMO about the diagnosis regime.

	**Morphology and Immunophenotype**	**Test Genetic**	**Radiographic Imaging**	**Prognosis**	**Refs.**
ESMO	Obligatory Diagnosis is usually possible through immunophenotyping peripheral blood only (III, A). LN biopsy and/or bone marrow biopsy may be helpful if immunophenotyping is not conclusive for the diagnosis of CLL (IV, A).	Del(17p), *TP53*mut, and IGHV status should be assessed before treatment (III, A). In the early and asymptomatic stage is not recommended (V, D).	It is not recommended in asymptomatic patients. Recommended for pulmonary symptomatic patients. Recommended before treatment with the BCL2 inhibitor to assess the tumor load and risk of tumor lysis syndrome.	Binet and Rai staging systems are relevant for treatment indication (III, A)	[58]
iwCLL	Obligatory	Molecular cytogenetics (FISH) for del(13q), del(11q), del(17p), and add (12) in PB lymphocytes. (Desirable). Conventional karyotyping in PB lymphocytes (Desirable). *TP53* mutation (needed to establish a prognostic profile in addition to the clinical staging). IGHV mutational status (needed to establish a prognostic profile in addition to the clinical staging). Serum β_2_-microglobulin (Desirable).	CT scan of chest, abdomen, and pelvis (Desirable) MRI and PET scans (NGI) Abdominal ultrasound (NGI)	Binet and Rai staging systems. CLL-IPI (Desirable).	[9]
ERIC	Obligatory	Strongly needed TP53 gene before starting the first and each subsequent line of treatment. Analyzing exons 4–10 is a minimal requirement with Sanger sequencing or NGS. Strongly needed to interpret IGHV mutational analysis before starting the first line of treatment. Alignment and determination of homology with PAGE or GeneScan.			[59,60]

CLL-IPI: CLL international prognostic index, NGI: not generally indicated, PAGE: Polyacrylamide gel electrophoresis, and PB: peripherical blood.

**Table 4:** Clinical studies of CIT use in CLL patients.

Clinical Study	Type of Study	Eligible Patients	Treatment	Endpoints	Conclusion	Ref.
FLAIR	Open label Randomized Phase III Controlled	Age between 18 and 75 years WHO performance status of 2 or lower Previously untreated CLL	Ibrutinib + rituximab (IR) vs. FCR	PFS	BR demonstrated a notable enhancement in PFS. It did not result in a significant improvement in OS.	[102]
CCL18	Multicenter Phase II Prospective Non-randomized	18 years old WHO performance status of 0 to 2 Life expectancy of at least 12 weeks Adequate renal and liver function	IR	Safety and efficacy of BR in previously untreated patients.	Apart from those with del(17p) who showed resistance to the treatment, the combination of BR is a safe and effective treatment for naïve CLL patients.	[103]
CCL10	Phase III Randomized Open label	Untreated fit patients with advanced CLL without del(17p)	FCR vs. BR	ORR	Smaller difference in median PFS between FCR and BR as well as no difference in OS.	[104]
ICLL-07-Filo	Phase II	≥18 years Binet stage C or Binet stage A and B with active disease. No prior treatment Absence of del(17p).	Obinutuzumab + ibrutinib followed by ibrutinib in patients achieving CR vs. FC-obinutuzumab in conjunction with ibrutinib.	PFS, OS, and minimal residual disease (MRD) in PB.	CIT with a set duration resulted in profound and lasting responses, leading to high survival rates. No distinctions were observed in the extent and persistence of MRD responses in PB based on the IGHV mutational status.	[105]

**Table 5:** Ibrutinib clinical studies in patients with CLL or SLL.

Clinical Study	Type of Study	Eligible Patients	Treatment	Endpoints	Conclusion	Refs.
RESONATE-2	Phase III, randomized, open label, multicenter.	CLL and small SLL patients (naïve or previously treated) and ineligible for purine analog therapy.	Ibrutinib vs. chlorambucil as first-line treatment	PFS, ORR, safety.	Ibrutinib demonstrated an extended OS. The extended RESONATE-2 data illustrate the advantages of initiating treatment with ibrutinib, even for patients exhibiting high risk.	[113,114]
PCYC-1102	Phase Ib/II study.	Patients receiving single-agent ibrutinib in first-line or r/r CLL/SLL.	Ibrutinib	Frequency and severity of AE	Ibrutinib demonstrated prolonged responses and sustained tolerability in first-line r/r CLL/SLL. Individuals who had a history of ≥4 prior therapies and those with del(17p) exhibited a higher frequency of progression.	[115]
ILLUMINATE	Phase III, randomized, open label.	Untreated CLL/SLL patients. Aged ≥ 65 or <65 years. At least one of the following conditions: cumulative illness rating score > 6, creatinine clearance < 70 mL/min, del(17p), or *TP53* mut.	Ibrutinib + obinutuzumab vs. chlorambucil + obinutuzumab as a first-line therapy	PFS	Ibrutinib + obinutuzumab for individuals with previously untreated CLL showed prolonged PFS compared to chlorambucil + Obinutuzumab.	[116]
E1912	Phase III, randomized.	CLL naïve patients aged 70 or less years.	Long-term efficacy ibrutinib + rituximab vs. FCR	PFS, OS	OS improvement in patients treated with ibrutinib + rituximab. This therapy provides better PFS compared to FCR in both IGHVm and IGHVum CLL patients.	[117]
ALLIANCE A041202	Phase III, randomized, open label.	Adults aged ≥ 65 years who had not received prior treatment for CLL.	BR vs. ibrutinib vs. ibrutinib + rituximab	PFS	Ibrutinib demonstrates superior PFS in older CLL patients compared to BR. However, the disparity in treatment duration complicates the comparison of AE.	[118,119]

**Table 6:** Acalabrutinib clinical studies in patients with CLL or SLL.

Clinical Study	Type of Study	Eligible Patients	Treatment	Endpoint	Conclusion	Refs.
ELEVATE-TN	Phase III, randomized, controlled.	Untreated patients aged ≥ 65 years.	Acalabrutinib + obinutuzumab, acalabrutinib or obinutuzumab + chlorambucil	PFS	Acalabrutinib, or acalabrutinib + obinutuzumab, demonstrated a significant enhancement in PFS compared to obinutuzumab + chlorambucil. Consider acalabrutinib monotherapy or in combination with obinutuzumab as treatment in naïve patients.	[123]
ASCEND	Phase III, randomized, open label, multicenter.	Patients aged ≥ 18 years diagnosed with CLL who had undergone at least one systemic therapy before.	Acalabrutinib, Idelasib + rituximab (idR) or BR	PFS	Meaningful enhancement in PFS when comparing acalabrutinib monotherapy to IdR or BR treatment regimens. Acalabrutinib demonstrated tolerability and profile. These results support the use of acalabrutinib monotherapy as a treatment for patients with r/r CLL, including those presenting high risk.	[124,125]
ELEVATE R/R	Phase III, randomized, international. multicenter, open label, non-inferiority.	Individuals who had undergone at least one previous therapy. ECOG ≤ 2 del(17) (p13.1) and/or del(11).	Acalabrutinib vs. ibrutinib	PFS	Acalabrutinib exhibited non-inferiority to ibrutinib in terms of PFS. There was a statistically significant reduction in the incidence of atrial fibrillation/flutter with acalabrutinib in patients with previously treated CLL. The incidence of hypertension was higher with ibrutinib.	[126]
MAJIC	Phase III, prospective, multicenter, randomized, open label.	Adults with naïve CLL/SLL meeting indication for treatment.	Acalabrutinib + venetoclax (AV) vs. VO	PFS	Evaluation to see if MRD-guided limited AV treatment is comparable to MRD-guided limited VO treatment in terms of PFS. The study is still in progress.	[127]

**Table 7:** Zanubrutinib clinical studies in patients with CLL or SLL.

Clinical Study	Type of Study	Eligible Patients	Treatment	Endpoints	Conclusion	Ref.
SEQUOIA	Phase III, open-label, multicenter study	Previously untreated CLL or SLL ≥65 or ≥18 years ECOG 0 -2	Zanubrutinib vs. BR	PFS	Zanubrutinib showed a notable improvement in PFS and safety profile compared to BR accompanied. These results lend support to zanubrutinib as a potential treatment option for untreated CLL and SLL.	[128]
ALPINE	Head-to-head Phase III study	r/r CLL/SLL patients	Zanubrutinib vs. ibrutinib	ORR PFS OS	Zanubrutinib demonstrated superiority over ibrutinib in terms of PFS, OS, and safety profile. In patients with del17p, TP53mut, or both, those treated with zanubrutinib experienced PFS compared with ibrutinib.	[129]
NCT03206918	Phase II, single-arm, multicenter study	r/r CLL/SLL patients	Zanubrutinib	ORR PFS	Results showed that administering zanubrutinib twice daily led to a significant occurrence of lasting responses. Zanubrutinib presents the possibility of enhanced safety and tolerability compared to current treatment choices.	[130]

**Table 8:** Venetoclax clinical studies in patients with CLL.

Clinical Study	Type of Study	Eligible Patients	Treatment	Endpoints	Conclusion	Refs.
CCL14	Phase III, multicenter, randomized, open label.	Untreated CLL ≥18 years.	VO vs. chlorambucil + Obinutuzumab.	PFS	2 years post-treatment discontinuation, the combination of VO demonstrated a significant enhancement of PFS compared to chlorambucil + obinutuzumab. In cases where the use of BTKi is not feasible due to potential AE, VO remains a reasonable choice.	[133,134]
CAPTIVATE	Phase II, randomized.	Previously untreated CLL patients age < 70 years.	3 cycles of ibrutinib, followed by 12 cycles of ibrutinib or ibrutinib + venetoclax. Confirmed undetectable MRD (uMRD): placebo or ibrutinib. Not confirmed uMRD: ibrutinib or ibrutinib + venetoclax.	1-year disease-free survival (DFS), uMRD status, and safety	The 95% 1-year DFS rate observed in patients randomly assigned to the placebo group, with confirmed uMRD, indicates the potential viability of a fixed-duration treatment using this all-oral, once-daily, chemotherapy-free regimen as a first line.	[135]
MURANO	Phase III, open label, randomized.	Patients with r/r CLL.	Venetoclax + rituximb (VR) vs. BR in patients with r/r CLL.	PFS, safety, and MRD status	VR treatment provides a long-lasting clinical response and confers a survival benefit compared to BR therapy.	[136]
GLOW	Phase III.	Patients aged ≤ 70 years with previously untreated CLL without del(17p) or tp53mut.	Fixed duration treatment ibrutinib + venetoclax.	Complete response (CR) rate, uMRD, PFS, OS, and safety	In older patients and/or those with comorbidities, the first-line treatment for CLL with ibrutinib + venetoclax exhibited superior PFS and achieved deeper and more enduring responses compared to chlorambucil–obinutuzumab.	[137]
CLL2-GIVe	Phase II, open label, multicenter.	Previously untreated patients with high-risk CLL and del(17p)/TP53 mut.	Triple combination of obinutuzumab + ibrutinib + venetoclax.	CR rate at cycle 15, PFS, and OS	After a median observation period of 38.4 months, the study found a 79.9% PFS and a 92.6% OS at 36 months. The research suggests that the CLL2-GIVe regimen is a hopeful fixed-duration first-line treatment for patients with high-risk CLL.	[138,139]

**Table 9:** Treatment recommendations in patients with del17 or tp53mut according to the main clinical guidelines.

Guideline	Drugs	Additional Information	Ref.
Spanish group of CLL (SGCLL)	Acalabrutinib Zanubrutinib Ibrutinib Ibrutinib + Venetoclax VO	Treatments are placed in order of recommendation.	[151]
Canadian Guideline	BTKi Ibrutinib Acalabrutinib	Favor ACAL for the best side effect profile. Indefinite therapy.	[152]
	VO	Improved PFS compared to CIT (chemoimmunotherapy). Less durable remission compared to BTKi. Finite therapy.	
Expert consensus on the management of CLL in Asia	BTKi Ibrutinib Acalabrutinib	Preferred first-line treatment of choice for patients with del17p or TP53 mutation. Patients who are intolerant to ibrutinib or who have relative contraindications to ibrutinib may still tolerate acalabrutinib. Second-generation BTKi, including acalabrutinib, may have a better safety profile than ibrutinib, especially in patients with high-risk disease characteristics.	[153]
	VO	BCL-2i can be considered in all CLL patients in need of therapy, including those with high-risk genomic features such as TP53 abnormalities.	
JAMA (Journal of the American Medical Association) First-line treatment	Indefinite treatment BTKi Acalabrutinib Ibrutinib Zanubrutinib	Second-generation BTKi (acalabrutinib and zanubrutinib) is preferred, given improved safety extrapolating from head-to-head trials in patients with relapse. Zanubrutinib had superior efficacy compared with ibrutinib.	[154]
	Fixed duration treatment VO	Consider continuation of venetoclax in patients with abnormal *TP53*, especially in patients with evidence of detectable disease at 12 months.	
ESMO	Ibrutinib or Acalabrutinib VO Venetoclax IdR	For the choice between VO versus ibrutinib or other BTKis, time-limited therapy would be preferred, but side effect profile and application mode must be considered.	[58]
German Society for Haematology and Medical Oncology (DGHO)	Acalabrutinib Zanubrutinib Ibrutinib	Continuous use of BTKi, mainly acalabrutinib or zanubrutinib, is preferred. If acalabrutinib or zanubrutinib are contraindicated or unavailable, ibrutinib (+/− obinutuzumab) remains a therapeutic option.	[155]
	VO		
	Ibrutinib + Venetoclax	Since August 2022, time-limited combination therapy (14 months) based on ibrutinib + venetoclax is also possible in the first line, which also includes patients with high-risk aberration. Based on the CAPTIVATE study.	

**Table 10:** Treatment recommendations in patients with no del17p or tp53 mut and IGHVm according to the main clinical guidelines.

Guideline	Type of Patient	Drugs	Additional Information	Ref.
SGCLL	Assess CIT scheme adapted to age and/or comorbidities (FCR/BR or Chlorambucil-O) when it is not possible to administer recommended treatment	Ibrutinib + Venetoclax VO	Treatments are placed in order of recommendation.	[151]
		Acalabrutinib Zanubrutinib		
		Ibrutinib		
Canadian Guideline	FIT patients	FCR	Longest remissions documented to date and possibility of cure. Finite therapy (only 6 months).	[152]
		VO	Highly effective therapy with very long remissions.	
		BTKi (Acalabrutinib)	Long remissions. Indefinite therapy (high cost).	
	UNFIT patients	VO	Preferred therapy. Finite therapy (only 12 months).	
		Acalabrutinib	Indefinite therapy. Very high cost.	
		CIT	Shorter remission than V-O. Finite duration therapy.	
Expert consensus on the management of CLL in Asia	FIT patients	BTKi	Fit patients < 65 years of age with IGHVm. Either FCR or other novel agents may be considered. Inform young patients about the risk of secondary malignancy and offer the option of CIT or novel agents (BTKi).	[153]
		CIT		
	UNFIT patients	VO	Both BTKi and BCL-2i have good clinical data to support their use.	
		BTKi		
ESMO	FIT patients	CIT: FCR or BR (patients > 65 years)	CIT is an alternative treatment used only if there is a reason for not using targeted therapies or when they are not available.	[58]
	UNFIT patients	VO CIT: Chlorambucil + Obinutuzumab, Ibrutinib, or Acalabrutinib		
*DGHO*	VO	If the genetic risk profile is favorable time-limited therapy with VO (12 cycles) should be preferred. If there are severe cardiac comorbidities, VO is primarily recommended.		[155]
	Acalabrutinib +/− Obinutuzumab	If renal function is impaired or if all-oral therapy is desired, primary therapy with a second-generation BTKi.		
	Zanubrutinib			
	Ibrutinib +/− Obinutuzumab	Higher cardiotoxicity of ibrutinib compared to second-generation BTKi. However, in the case of renal failure, preference should be given a BTKi.		
	Ibrutinib + Venetoclax	Can be used in intermediate-risk patients (IGHVum) as a temporary therapy (15 cycles).		

**Table 11:** Treatment recommendations in patients with no del17p or tp53mut and IGHVum according to the main clinical guidelines.

Guideline		Type of Patient			Drugs	Additional Information				Ref.
SGCLL		Acalabrutinib Ibrutinib + Venetoclax VO Zanubrutinib Ibrutinib				Same level of recommendation. To be evaluated according to patient profile.				[151]
Canadian Guideline		FIT patients			Acalabrutinib	Better PFS (elevate R/R study) than Ibrutinib. Conflicting OS				[152]
					VO	Less PFS than BTKi				
		FCR Ineligible			Acalabrutinib	Improved PFS compared to CIT.				
					VO	Effective therapy expected to provide several years of treatment-free duration. Finite duration (12 months)				
					Acalabrutinib–Obinutuzumab	Improved OS compared to CIT.				
Expert consensus on the management of CLL in Asia		FIT patients			BTKi	Could be used in preference to CIT. Lower toxicity.				[153]
					CIT	Can be used as a first-line treatment option.				
		UNFIT patients			VO					
					BTKi	May be considered the preferred first-line treatment of choice for this patient.				
ESMO		FIT patients			Ibrutinib or CIT: FCR or BR (>65 years patients)					[58]
		UNFIT patients			VO, Ibrutinib–Acalabrutinib, CIT: Chlorambucil–Obinutuzumab					
DGHO		Acalabrutinib +/− Obinutuzumab Zanubrutinib Ibrutinib +/− Obinutuzumab			Some studies (ALLIANCE and ILLUMINATE) with BTKi showed reduced PFS in the IGHVum group. Due to the cardiovascular toxicity profile, therapy with ibrutinib is primarily not recommended unless patients are young, fit, and have no prior cardiac disease.					[155]
		VO			Temporary therapy (12 cycles). CCL14 showed a significant difference in PFS in patients with unmutated IGHV status. However, the result of the CLL17 study on whether VO is inferior to long-term treatment with BTKi (including non-mutated patients) is still pending.					
		Ibrutinib + Venetoclax			Data from the GLOW study show shorter PFS for IGHVum at short follow-up for this group compared to the subgroup with IGHVm.					
NCCN/iwCLL [13,156]	Front-line therapy	Acalabrutinib +/− obinutuzumab VO Zanubrutinib				These options are recommended across almost every subgroup of patients.				
	Clinical trial available	Yes	With no clear evidence of a functional cure, it is important to enroll patients in clinical trials when available.							
		No	Check IGHV status	Mutated	Check CLL FISH	Only del 13q+	Age < 65	Yes	Discuss/consider FCRx6. This regimen is not preferred in the current era of targeted therapies for CLL and SLL.	
								No	Check FISH/tp53 mutations.	
				Unmutated	Check CLL FISH/tp53 mutation.	del17+ or tp53mut	Acalabrutinib		Renal insufficiency. Extensive infections (except in cases of aspergillosis).	
							Zanubrutinib			
						Others	Consider comorbidities.	VO (12 mo)	Atrial fibrillation or hypertension. Need for anticoagulation. Preference for time-limited therapy.	

## Data Availability

Not applicable.
